# Maternal Infection Is a Risk Factor for Early Childhood Infection in Filariasis

**DOI:** 10.1371/journal.pntd.0003955

**Published:** 2015-07-30

**Authors:** Madhusmita Bal, Prakash K. Sahu, Nityananda Mandal, Ashok K. Satapathy, Manoranjan Ranjit, Shatanu K. Kar

**Affiliations:** Division of Immunology, Regional Medical Research Center (Indian Council of Medical Research), Chandrasekharpur, Bhubaneswar, Odisha, India; Emory University, UNITED STATES

## Abstract

**Background:**

Global Program to Eliminate Lymphatic Filariasis (GPELF) launched by WHO aims to eliminate the disease by 2020. To achieve the goal annual mass drug administration (MDA) with diethylcarbamazine (DEC) plus albendazole (ABZ) has been introduced in all endemic countries. The current policy however excludes pregnant mothers and children below two years of age from MDA. Since pregnancy and early childhood are critical periods in determining the disease outcome in older age, the present study was undertaken to find out the influence of maternal filarial infection at the time of pregnancy on the susceptibility outcome of children born in a community after implementation of MDA for the first time.

**Methodology and Principal Findings:**

The participants in this cohort consists of pregnant mothers and their subsequently born children living in eight adjacent villages endemic for filarial infections, in Khurda District, Odisha, India, where MDA has reduced microfilariae (Mf) rate from 12% to 0.34%. Infection status of mother and their children were assessed by detection of Mf as well as circulating filarial antigen (CFA) assay. The present study reveals a high rate of acquiring filarial infection by the children born to infected mother than uninfected mothers even though Mf rate has come down to < 1% after implementation of ten rounds of MDA.

**Significance:**

To attain the target of eliminating lymphatic filariasis the current MDA programme should give emphasis on covering the women of child bearing age. Our study recommends incorporating supervised MDA to Adolescent Reproductive and Sexual Health Programme (ARSH) to make the adolescent girls free from infection by the time of pregnancy so as to achieve the goal.

## Author Summary

Lymphatic filariasis, commonly known as elephantiasis, is a painful and profoundly disfiguring disease. A massive global effort has been undertaken to eliminate this disease by 2020 using annual mass drug administration (MDA). However the MDA excludes pregnant women and children below two years of age, which are the two critical periods thought to be playing a major role in determining the disease susceptibility in older age. We have been working on the impact of filarial infection at the time of pregnancy on neonates since 2009 in India. We have assessed the impact of maternal infection among a group of children from the time of their birth till they attain early childhood. We have observed that children born to mothers having filarial infection at the time of pregnancy were more prone to infection at early childhood than born to infection free mother in an area having very low infection rate after MDA. Our finding reveals that infection in mother at the time of pregnancy plays a crucial role in acquiring infection in children. Hence to achieve the goal of elimination of filariasis the women of child bearing age should be treated with antifilarials through MDA under supervision.

## Introduction

Lymphatic filariasis (LF) is the second leading cause of chronic disability worldwide. According to recent estimate around 120 million people have been infected with LF in 73 countries and more than 1.1 billion (20% of the world’s population) are at risk of acquiring infection [1]. Two-thirds of the endemic population resides in South-East Asia and one-third lives in India [2]. Out of 30 states in India, seven states namely Andhra Pradesh, Bihar, Kerala, Odisha, Uttar Pradesh, Tamil Nadu, and West Bengal contribute over 86% of Mf and 97% of disease cases in the country [3].

In 1997, WHO and its member states made a commitment to eliminate LF as public health problem by 2020 through World Health Assembly Resolution WHA 50.29. The National Health Policy (2002) has set the goal of elimination of LF in India by 2015 by annual mass drug administration (MDA). Odisha, an eastern Indian state, has experienced 10 rounds of MDA since 2004. But according to NVBDCP (National Vector Borne Disease Control Programme, India) though the Mf rate has come down from 2.6% to 0.34% during this time yet complete elimination of filariasis has not been achieved [4]. The concept of MDA includes administration of single dose of DEC (6mg/kg body weight) plus albendazole (400mg) to everybody in the community. Since DEC can cause anaphylactic reactions pregnant women, children below two years of age and persons who are very sick from other illness are not covered under MDA. When a proportion of the population fails to comply with MDA, a potential reservoir for the parasite is left untreated, opening the door to recrudescence or to potential risk factors for increasing the susceptibility status of new born and thus reducing the probability of the program’s success. Our previous studies have shown that transplacental transfer of circulating filarial antigen (CFA) can lead to in-utero sensitization and immune-modulation in neonates born to filarial infected mother [5–7]. Hence question arises whether the sensitization of the foetus and immune-modulation at the time of delivery can ultimately influence the disease outcome in children during their natural exposure to filariasis in MDA ongoing area, where the Mf rate has come down to below threshold level (<1%). Therefore the present investigation was undertaken to find out the influence of maternal infection on the susceptibility outcome of children born after implementation of MDA for the first time and its implication on the success of the current elimination programme.

## Materials and Methods

### Ethics statement

The human ethical committee of Regional Medical Research Centre, Bhubaneswar has approved the study and recommended to obtain oral consent from the study participants. All enrolled mothers have been explained about the purpose of study in local language in presence of an impartial witness of the community like Auxiliary Nurse Midwife (ANM) / Accredited Social Health Activist (ASHA) / Anganwadi Workers (AWW). All the enrolled mothers have given face to face consent to participate in the study for themselves and their children as well without a sign consent form. The name and detailed address of the participants who have given consent was recorded in our data sheet at the time of enrolment for tracking during follow-up. The oral consent was preferred because of linguistic or literacy demands of the written format.

### Study area

The district Khurda situated on the coast of the Bay of Bengal is one of the highly endemic districts for LF [8] and has experienced 10 rounds of MDA with > 85% coverage. The district has reported Mf rate of 0.34% in 2013 compared to 12% in 2004. Currently, around 1.8 million people are at risk of filarial infection in the district. Amongst them 51.91% lives in rural areas (total 1358 villages) and 48.09% in urban areas (total 3 municipalities and 2 notified area councils).

### Study design and study participants

This is a hospital based cohort study conducted in eight adjacent villages of Bajapur Panchayat of Khurda block-a highly endemic area for filariasis (*Wuchereria bancrofti*). Women admitted in O&G Department of Khurda District Headquarter Hospital for delivery from July 2009–July 2011 and are permanent residents of these villages have been enrolled for the study conveniently. Mother-infant pairs were excluded from the study in case of i) complicated infant delivery resulting in significant infant morbidity at birth, ii) premature delivery, iii) mother who had known chronic illness or iv) the family who had plans to relocate after delivery. Upon enrolment, mothers underwent a detailed questionnaire that queried their age, parity, education level, clinical history of filariasis and history of drug consumption in MDA. None of the mothers had signs/symptoms of clinical filariasis at the time of admission. Based on their statement all the enrolled mothers have consumed the antifilarials distributed during MDA before pregnancy. Paired cord and maternal blood samples were collected at the time of uncomplicated delivery. Venous blood samples were collected from mothers before delivery. Venous umbilical cord blood samples from neonates were collected immediately after birth. The collection of cord blood involved direct aspiration via puncture of the ethanol-sterilized umbilical vein at a site distal to the placenta, to reduce minimum cross-contamination. Maternal and cord blood samples were collected in different sized tubes to avoid the chance of mislabelling. Sera were stored at -70°C till further use.

The children born full-term, healthy and whose mothers agreed to continue participation have been enrolled during follow up. Out of 158 mother-newborn pairs enrolled during 2009–2011, 63.9% (101/158) could be followed up along with their children during house to house visit in 2014. At the time of follow-up the mothers averaged 29.2 years of age, and most of them had a primary school education. Majority of the enrolled mothers (81%) identified ‘‘Homemaker/Housekeeper” as their primary occupation. The physical assessment of mothers and children were conducted by a physician and examined for presence of Mf. Venous blood samples (1ml) were collected from enrolled mothers and their children along with detailed clinical history.

### Maternal and children infection status

*W*. *bancrofti* infection was detected in mother and their children either by detection of Mf in thick blood smear of peripheral blood collected at night between 20:30 and 22:30 by microscopy or by detection of CFA in serum samples using commercially available Og4C3 antigen detection assay kit (Trop BioMed, Townsville, Australia).

### Statistical analysis

The statistical analysis was done using GraphPad *Prism* software. Differences in proportions between the two groups were assessed using the Fisher exact test and the power of the study was measured using post hoc power analysis. The level of significance was set at 0.05

## Results

**Fig 1:**
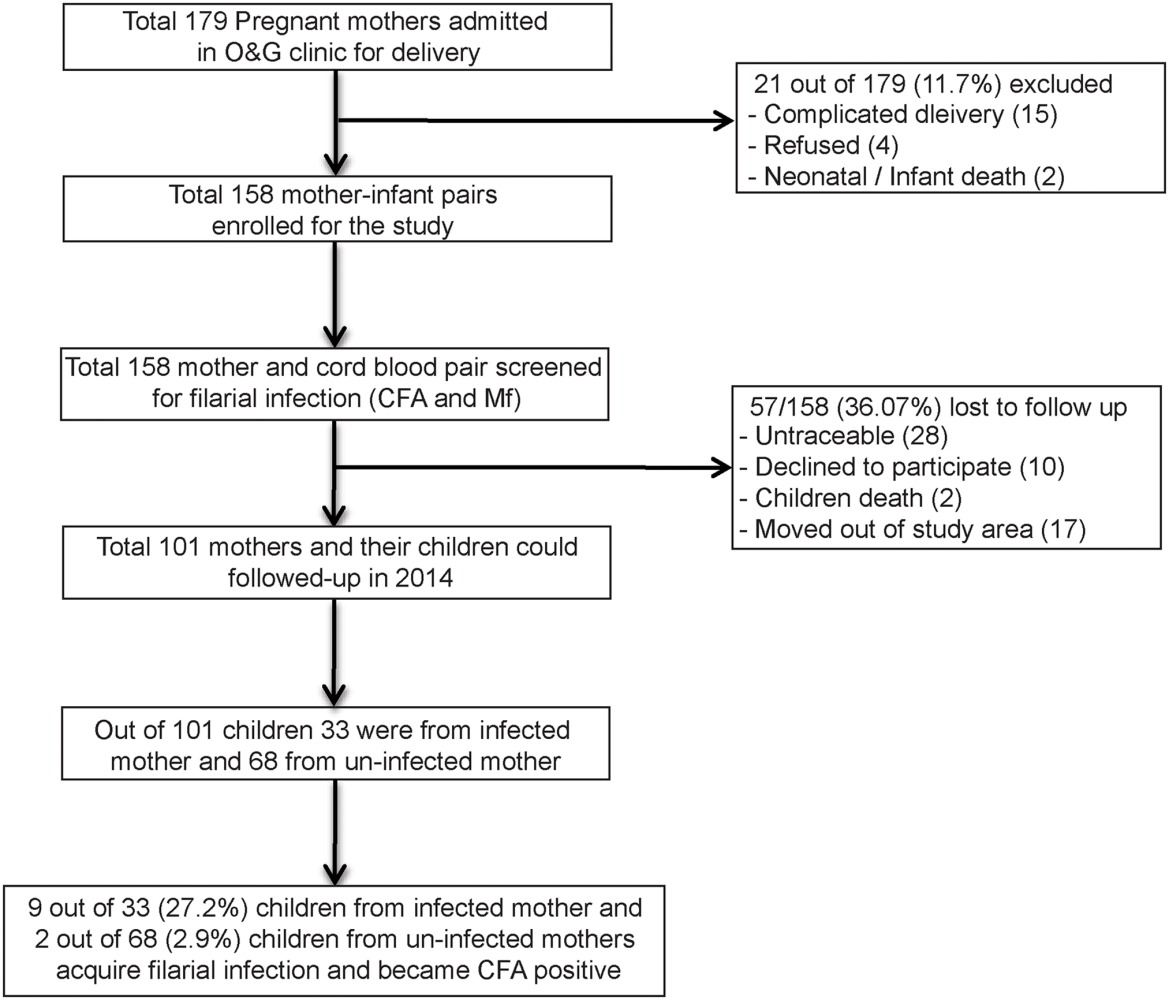
The flow diagram of the cohort study.

An overview of the enrolment and follow up of participants is presented in Fig 1. A total number of 179 pregnant women admitted to O& G clinic for delivery from July 2009 to July 2011were selected for the study. Of them 21 (11.7%) mothers were excluded because of complication during delivery or infant death or unwillingness. None of the mothers have been enrolled more than once. Finally 158 mother-new born pairs were enrolled for the study. Amongst them 24% of the mothers have multiparity status. As would appear from the baseline characteristics about 45% of the mothers were positive for CFA (GM: 1925, range: 630–16596) and 11.8% for Mf (3–210 per 60μl blood). The transplacental transfer of CFA from mother to cord was detected in 15.8% of the infected mother, while none of the cord blood was found to be positive for Mf.

Out of 158 mother-new born pairs a total of 101 pairs have been examined during the follow-up. Amongst them 33 children were found to born from mothers who had filarial infection (MF+ve /CFA+ve: 4 and Mf -ve / CFA +ve: 29) at the time of delivery and 68 from filarial uninfected mother(CFA-ve and Mf-ve). Out of 33 infected mothers, 18 mothers are still harbouring filarial infection (CFA +ve but Mf -ve), 5 mothers have cleared CFA but developed acute symptoms of filariasis and 10 have cleared CFA without developing any clinical signs/symptoms of filariasis. The geometric mean (GM) of CFA levels of the mothers at the time of follow-up was 232units (range: 128–7762) compared to 2125 (range: 930–16596) at the baseline of the study. All mothers were free from Mf at the time of follow-up compared to 12.1% (4/33) at the time of enrolment. Interestingly all 68 uninfected mothers maintained infection free status at the time of follow-up.

Out of 33 children born to infected mothers 9 (27.2%) have acquired filarial infection and became CFA positive (GM: 147, range: 128–230), while 6 out of these 9 children belongs to mothers (n = 15) who are CFA negative during follow up. Moreover amongst these 6 children 3 belongs to mothers who have developed clinical filariasis during follow up and rest 3 belongs to mothers who are free from CFA and clinical signs/symptoms. In contrast 2.9% (2/68) of the children born to uninfected mothers have become CFA positive (GM: 133) during this period. None of the children had developed either any clinical signs/symptoms of filariasis or Mf in their peripheral blood (Table 1).

**Table 1:** Status of filarial infection among the children born to filarial infected and uninfected mother in a MDA ongoing area of Odisha.

Status of Mother								Present status of follow-up children (2014)						
Baseline (2009)				Follow-up (2014)										
CFA status	N	CFA unit GM* (range)	Mf +ve	CFA status	N	CFA units GM* (range)	Mf +ve	N	Mean age (years)	Mf +ve	Clinical symptom	CFA positive	% CFA +ve	CFA GM* (range)
CFA +ve	33	2125 (930–16596)	4	+ve	18	232 (128–7762)	0	33	4.2	0	No	9	27.2	147 (125–230)
CFA-ve	68	0	0	-ve	68	0	0	68	4.3	0	No	2	2.9	133

*GM: Geometric mean

Comparison of data shown that there is an extremely significant association (P = 0.0006, 95% confidence interval = 0.01628 to 0.4011, power of the study: 92.2%) between infection status of mother and acquiring of infection by the children born to them.

## Discussion

For the first time our study has shown the impact of in- utero exposure to filarial infection on susceptibility outcome in early childhood during their natural exposure to filariasis in a MDA ongoing area, where the Mf rate has come down to below threshold level. This study further reveals that though a proportion of infected mothers have cleared infection during follow-up period due to ongoing MDA, yet children born to them acquire infection similarly as the children born to mothers who have not cleared the infection.

Fetal adaptive immune responses are common in neonates who have been exposed to maternal infection during pregnancy but not infected themselves. Such responses could affect the development of immunity to the homologous pathogens and their control during the first few years of life [9]. Pregnancy and early childhood are critical periods in which the inherited immune system of a child is shaped by the environment both in-utero and soon after birth. This in turn is thought to determine the disease outcome in older age [10]. Recently studies have shown that in-utero exposure to helminths can non-specifically modulate the offspring’s susceptibility to inflammation mediated diseases [11]. This might affect the development of fetal immunity and susceptibility to postnatal infections independently of in-utero transmission of the pathogens [9]. A few cross sectional studies in filariais have shown that children born to infected mothers are more susceptible to infection than children born to uninfected mother [12 13]. Moreover some other studies including our own have demonstrated transplacental transfer of filarial antigen and in-utero sensitization that modulates immune responses in neonatal stages, where an increased levels of IL-10 and decreased levels of interferon gamma (IFN-γ) were observed in cord blood of infected mothers. A positive correlation of IFN-γ with IgG3 and IL-10 with IgG4 and CFA, indicates modulation of immune responses in cord bloods of sensitized foetus [5–6,13]. It is also observed that in-utero acquisition of T cell immunity to filarial antigens can influence the susceptibility to *W*. *bancrofti* infection during childhood [14]. Further epidemiological evidence suggests that in-utero sensitisation results in down-regulated responses among the offspring, on encountering the homologous antigen. This may be due to a bias in the foetal and neonatal immune response towards the development of T regulatory and T-helper (Th)17 responses rather than Th1 responses[11]. All these observations substantiate our present finding which demonstrates that children born to mothers who had filarial infection at the time of pregnancy are acquiring filarial infection significantly more than children born to infection free mother because of in-utero sensitisation. Even the children born to mothers who were CFA positive initially but subsequently have cleared CFA and / or devolved clinical signs/symptoms have been found to acquire infections similar to the children born to mothers who are continuing to be CFA positive. In addition since Mf is necessary for filarial transmission, it can be presumed that children living in the household with at least one infected carrier (the mother) are at higher risk of infection than children living in household of an uninfected mother. But in the present study only one children is born to the mother who had microfilaraemia at the time of pregnancy indicating that shared household is not playing any major role towards the susceptibility of children born to infected mothers. Though all the children are living in the same endemic area and have equal exposure to infection still the susceptibility status observed to be dependent on the infection status of the mother during pregnancy. Hence we hypothesize that immunomodulation due to in-utero sensitisation is playing a crucial role towards outcome of the disease in their early childhood.

Mathematical models predict that at least four to six rounds of annual mass treatment with a coverage rate of 65% is required for the elimination of LF, assuming that the reproductive life span of *Wuchereria bancrofti* is five years [15,16]. Though according to NVBDCP the coverage rate of MDA in our study area was more than 85% and the Mf rate has come down to below threshold level after 6 rounds of MDA, yet we have observed11.8% of the pregnant mothers to be Mf+ve, while the overall Mf prevalence in the population was 0.49% in the same study area during initiation of the study (4,5). The high prevalence of infection in pregnant mothers might be due to back-to-back pregnancy which inhibits these mothers to receiving of MDA for longer period of time or might be due noncompliance to MDA. Hence exclusion of pregnant mothers from MDA not only serves as the potential reservoir of filarial infection but also plays a key role in acquiring new infections by the new born children. Besides children and pregnant mothers residual microfilaremics (individuals having Mf in their circulation after several rounds of MDA) might be another possible factor for persistence of infection in the MDA ongoing area, since *W bancrofti* has no other natural animal reservoirs. The obvious limitation of our study is small sample size (positive mothers). The second major limitation is that the mothers enrolled in the study were not under supervised MDA. But we have analysed the data assuming that they have consumed the drug distributed during MDA based on their statement before pregnancy and during follow up.

### Conclusion

We can conclude that there is a strong association between maternal filarial infection and the infection outcome in children born to them. This might be one of the reasons for persistence of filarial infection among the children < 5 years even though Mf rate has come down to below threshold level in this endemic area after implementation of 10 rounds of MDA. Hence the present finding reinforces the importance of implementation of more efficient prevention strategies of lymphatic filariasis in pregnant women and their children. In order to achieve the target of eliminating lymphatic filariasis by 2020 globally and by 2015 in India the current MDA programme can include supervised MDA therapy in to the current Adolescent Reproductive and Sexual Health Programme (ARSH) so as to make the women of child bearing age free from infections before pregnancy, because it has been observed that continuous reduction of CFA level after repeated treatments can eliminate *W bancrofti* infection.

## Supporting Information

S1 Checklist STROBE checklist. (DOCX)
