# Supplementary material for: Maternal Infection Is a Risk Factor for Early Childhood Infection in Filariasis
Source: PLoS Negl Trop Dis. 2015 Jul 30;9(7):e0003955. doi: 10.1371/journal.pntd.0003955 (PMC4520468; doi:10.1371/journal.pntd.0003955)
Supplement: S1 Checklist — (DOCX) [file pntd.0003955.s001.docx]

**STROBE STATEMENT**

|  | Item No | Recommendation |
| --- | --- | --- |
| **Title and abstract** | 1 | (*a*) Maternal infection is a risk factor for early childhood infection in filariasis |
|  |  | (*b*)The WHO has targeted to eliminate Lymphatic filarisis globally by 2020 by annual mass drug administration (MDA).The current policy however excludes pregnant mothers and children below two years of age from MDA. Since pregnancy and early childhood are critical periods in determining the disease outcome in older age, the present study was undertaken to find out the influence of maternal filarial infection at the time of pregnancy on the susceptibility outcome of children born in a community after implementation of MDA for the first time. The study reveals that children born to infected mother are at higher risk of acquiring filarial infection than the children born to uninfected mothers even though Mf rate has come down to < 1% after implementation of ten rounds of MDA. Our study recommends incorporating supervised MDA to Adolescent Reproductive and Sexual Health Programme (ARSH) to make the adolescent girls free from infection by the time of pregnancy so as to achieve the goal. |
| Introduction | | |
| Background/rationale | 2 | Lymphatic filariasis (LF) is the second leading cause of chronic disability worldwide. In 1997, WHO and its member states made a commitment to eliminate LF as public health problem by 2020 through World Health Assembly Resolution WHA 50.29. ). However the MDA excludes pregnant women and children below two years of age, since DEC can cause anaphylactic reactions in some cases. When a proportion of the population fails to comply with MDA, a potential reservoir for the parasite is left untreated, opening the door to recrudescence or to potential risk factors for increasing the susceptibility status of new born and thus reducing the probability of the program’s success. Our previous studies have shown transplacental transfer of circulating filarial antigen (CFA) that leads to in-utero sensitization and immune-modulation in neonates born to filarial infected mother. Hence question arises whether the sensitization of the foetus and immune-modulation at the time of delivery can ultimately influence the disease outcome in children during their natural exposure to filariasis in MDA ongoing area, where the Mf rate has come down to below threshold level (<1% ). Therefore the present investigation was undertaken to find out the influence of maternal infection on the susceptibility outcome of children born after implementation of MDA for the first time and its implication on the success of the current elimination programme. |
| Objectives | 3 | To investigate the influence of maternal filarial infection in susceptibility outcome of children born to them |
| Methods | | |
| Study design | 4 | This is a hospital based cohort study |
| Setting | 5 | Women admitted in O&G Department of Khurda District Headquarter Hospital, Odisha(India) for delivery from July 2009–July 2011 and are permanent residents of eight adjacent villages of Bajapur Panchayat of Khurda block-a highly endemic area for filariasis (*Wuchereria bancrofti*) have been enrolled for the study conveniently. |
| Participants | 6 | (*a*) The study participants are pregnant women residing permanently in MDA ongoing area and children born to them subsequently. The eligibility criteria includes uncomplicated/ premature delivery, free from chronic illness, do not have the plan to relocate after delivery and willing to participate. The children born full-term, healthy and whose mothers agreed to continue participation have been enrolled during house to house visit for follow up. |
|  |  | (*b*) The infection status of children born to 33 infected mothers are matched with 68 children born to uninfected mothers |
| Variables | 7 | (i) Mf / CFA status of mother during enrolment and follow up, (ii) ) Mf / CFA status of cord blood and (iii) Mf/CFA status of children born to them. Diagnosis of Mf was done in thick blood smear of peripheral blood collected at night between 20:30 and 22:30 by microscopy and detection of CFA in serum samples using commercial Og4C3 antigen detection assay |
| Data sources/ measurement | 8* | The data generated by examination of blood collected directly from the participants. Paired cord and maternal blood samples (1ml) were collected at the time of uncomplicated delivery. Venous blood samples were collected from mothers before delivery. Venous umbilical cord blood samples from neonates were collected immediately after birth. The collection of cord blood involved direct aspiration via puncture of the ethanol-sterilized umbilical vein at a site distal to the placenta, to reduce minimum cross-contamination. Maternal and cord blood samples were collected in different sized tubes to avoid the chance of mislabelling. During follow up venous blood samples (1ml) were collected from enrolled mothers and their children along with detailed clinical history. |
| Bias | 9 | Only the permanent residents of the study area and admitted in the hospital during delivery has been enrolled to avoid any sampling bias |
| Study size | 10 | All eligible pregnant women in the study area has been enrolled |
| Quantitative variables | 11 | Quantitative variables of CFA level were analysed using appropriate parametric tests |
| Statistical methods | 12 | The statistical analysis was done using GraphPad Prism software. Differences in proportions between the two groups were assessed using the Fisher exact test and the power of the study was measured using post hoc power analysis. The level of significance was set at 0.05 |
| Results | | |
| Participants | 13* | (a) During 2009-2014 total 179 pregnant mothers were admitted in O&G clinic for delivery. Out of them total 158 mother-infant pairs enrolled for the study based on their eligibility criteria. During 2014 total 101 mothers and their children could followed-up and analysed. |
|  |  | (b) At the time of enrolment 21 out of 179 were excluded because of complicated delivery, refused to participate and neonatal / infant death and at the follow up 57 out of 158 enrolled participants were lost to follow-up because of untraceable, declined to participate, children death and moved out of study area. |
|  |  | (c) A flow diagram of the participants has been provided in the text of the paper |
| Descriptive data | 14* | (a) The study participants were pregnant mothers (n=158) averaged 24.7 years of age during enrolment. Amongst them 24 % of the mothers have multiparity status. They were living in eight highly filarial endemic rural villages of Odisha (India) and most of them had a primary school education. Majority of the enrolled mothers (81 %) identified ‘‘Homemaker/Housekeeper’’ as their primary occupation. At the time of enrolment 11.8% of the mothers were Mf+ve and 45% of the mothers were positive for CFA. |
|  |  | (b) No participants with missing data |
|  |  | (c) Follow up was done after 5 years of enrolment |
| Outcome data | 15* | Out total children 27.2 % of children born to infected mothers and 2.8% of uninfected mothers have acquired filarial infection |
| Main results | 16 | Out of 158 mother-new born pairs a total of 101 pairs have been examined during the follow-up. Amongst them 33 children were found to born from mothers who had filarial infection (MF+ve /CFA+ve: 4 and Mf –ve / CFA +ve: 29) at the time of delivery and 68 from filarial uninfected mother(CFA-ve and Mf-ve). Out of 33 infected mothers, 18 mothers are still harbouring filarial infection (CFA +ve but Mf –ve ), 5 mothers have cleared CFA but developed acute symptoms of filariasis and 10 have cleared CFA without developing any clinical signs/symptoms of filariasis. The geometric mean (GM) of CFA levels of the mothers at the time of follow-up was 232units (range: 128-7762) compared to 2125 (range: 930 - 16596) at the baseline of the study. All mothers were free from Mf at the time of follow-up compared to 12.1% (4/33) at the time of enrolment. Interestingly all 68 uninfected mothers maintained infection free status at the time of follow-up.  Out of 33 children born to infected mothers 9 (27.2%) have acquired filarial infection and became CFA positive (GM: 147, range: 128-230), while 6 out of these 9 children belongs to mothers (n=15) who are CFA negative during follow up. Moreover amongst these 6 children 3 belongs to mothers who have developed clinical filariasis during follow up and rest 3 belongs to mothers who are free from CFA and clinical signs/symptoms. In contrast 2.9% (2/68) of the children born to uninfected mothers have become CFA positive (GM: 133) during this period. None of the children had developed either any clinical signs/symptoms of filariasis or Mf in their peripheral blood  Comparison of data shown that there is an extremely significant association (P=0.0006, 95% confidence interval= 0.01628 to 0.4011, power of the study: 92.2%) between infection status of mother and acquiring of infection by the children born to them. |
| Other analyses | 17 | No other analysis has been done |
| Discussion | | |
| Key results | 18 | *W bancrofti* infection rate was found to be significantly high among the children born to mothers who had filarial infection at the time of pregnancy than the children born to infection free mother |
| Limitations | 19 | The obvious limitation of our study is small sample size (positive mothers). The second major limitation is that the mothers enrolled in the study were not under supervised MDA. Hence we have analysed the data assuming that they have consumed the drug distributed during MDA based on their statement before pregnancy and during follow up. |
| Interpretation | 20 | High rate of infection among the children born to mothers infected with *W bancrofti* during pregnancy might be one of the reasons for persistence of filarial infection among the children < 5 years even though Mf rate has come down below threshold level(<1%) in this endemic area after implementation of 10 rounds of MDA. |
| Generalisability | 21 | The results can be generalized beyond the immediate study since many cross sectional studies carried out in other countries have shown that maternal infection is a risk factor for childhood infection. |
| Other information | | |
| Funding | 22 | Indian Council Of Medical Research, New Delhi |
